# Determination and Validation of Standard Enthalpies of Formation and Sublimation of Potassium Salts Using Solution Calorimetry and Quantum‐Chemical Calculations

**DOI:** 10.1002/open.202500502

**Published:** 2026-02-08

**Authors:** Zohreh Amanollahi, Dzmitry H. Zaitsau, Karsten Müller, Olga S. Bokareva, Riko Siewert

**Affiliations:** ^1^ Leibniz Institute for Catalysis (LIKAT) Rostock Germany; ^2^ Department Life, Light & Matter of the Faculty of Interdisciplinary Research University of Rostock Rostock Germany; ^3^ Institute of Technical Thermodynamics University of Rostock Rostock Germany; ^4^ Institute of Chemistry University of Rostock Rostock Germany

**Keywords:** density functional theory, lattice energy, reaction enthalpy, solution enthalpy

## Abstract

This work aims to develop an approach for the systematic determination of the formation and sublimation enthalpies of inorganic compounds using the examples of potassium bicarbonate, potassium carbonate, and potassium formate. The standard enthalpies of formation in the solid and gas phases are determined using solution calorimetry and the G4 method, respectively, while the enthalpies of sublimation are calculated from lattice energies. Density functional theory (DFT) calculations at the PBE‐D3/projector‐augmented‐wave level are well‐suited to determine sublimation enthalpies based on lattice energies and thus to validate the standard enthalpies of formation of inorganic compounds in the solid and gas phases. The uncertainty associated with the determination of standard formation enthalpies in the solid phase using solution calorimetry is roughly 0.5 kJ·mol^−1^, while the uncertainty of sublimation enthalpies from DFT calculations is approximately less than 10 kJ·mol^−1^, consistent with recent benchmark studies on representative molecular test sets. Given that the determination of sublimation enthalpies for salts based on vapor pressures is currently not feasible with existing techniques, DFT calculations are a promising approach for determining this quantity. In conclusion, the combination of solution calorimetry and quantum‐chemical calculations offers a consistent framework for determining key thermodynamic properties of inorganic salts.

## Graphical abstract

The graphical abstract is structured into three horizontal sections, each presenting a key concept on the left and a corresponding molecular‐scale illustration on the right. The top section shows crystal lattice models of potassium salts and conveys the solid‐state structural motifs underlying the computational approach. The middle section presents the reaction equation for the potassium formate/potassium bicarbonate equilibrium and frames this reversible reaction as a pathway for hydrogen uptake and release. The bottom section depicts potassium formate and potassium bicarbonate surrounded by hydration shells, illustrating aqueous solvation and indicating how solvation can modulate the reaction enthalpy associated with hydrogenation in solution. Together, the scheme summarizes how experimental solution calorimetry and quantum‐chemical calculations can be combined to obtain a consistent set of thermodynamic quantities for inorganic salts, including formation and sublimation enthalpies.

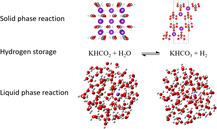

## 1 Introduction

The determination of standard enthalpies of formation and enthalpies of sublimation of solid organic compounds is a well‐established experimental procedure. To determine the standard enthalpies of formation of organic compounds, static combustion calorimetry is the most commonly used method [1]. However, this method is not suitable for metal‐containing compounds. For the application of combustion calorimetry, a rotating bomb would be required, along with additional study of the final composition and introduction of corresponding nonobvious corrections. Unfortunately, no precise commercial devices of this type are available, and only very few research groups in the world have access to this technique that can provide precise experimental values.

**FIGURE 1:**
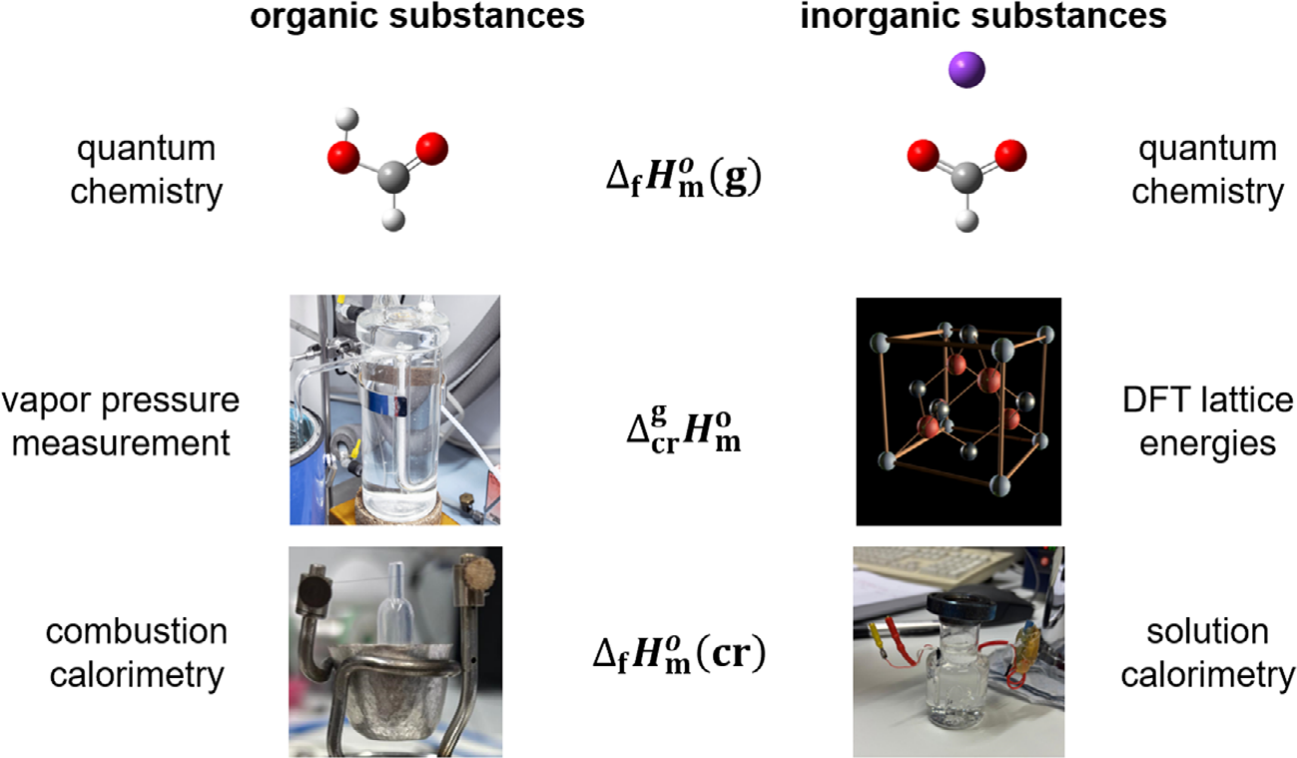
Determination and validation of enthalpies of formation and sublimation for organic substances (left side) and inorganic substances (right side).

The enthalpies of sublimation are typically determined using temperature‐dependent vapor pressure measurements or by direct calorimetric determination of the heat absorbed by a sample during evaporation. The commonly used methods are available for measuring vapor pressures of organic compounds in the range from ≈10^−3^ Pa to 10^6^ Pa [2, 3, 4]. For very low volatile compounds such as ionic liquids, the quartz crystal microbalance method enables the measurement of the vapor pressure in the range of 10^−6^ Pa [5, 6]. Sublimation enthalpies of inorganic salts can, in principle, be obtained from vapor pressure measurements. This possibility has been demonstrated for salts such as NaCl [7]. However, obtaining reliable vapor pressure data for inorganic salts at room temperature remains challenging. Measurements at elevated temperatures are possible, but the vapor pressures of most salts at ambient conditions are far too low to be determined accurately. In addition, experimental complications and uncertainties in the gas phase composition further limit the applicability of this method at the reference temperature of 298.15 K. For these reasons, sublimation enthalpies at 298.15 K cannot be determined reliably for many inorganic salts based on vapor pressure measurements. To address these limitations, we present an alternative approach for determining sublimation enthalpies of inorganic salts. By combining solution calorimetry with density functional theory (DFT) calculations, this study presents a method for determining the standard formation and sublimation enthalpies of inorganic salts, similar to the approach commonly used for organic compounds (see Figure 1).

DFT has become an indispensable tool for predicting the thermodynamic properties of crystalline materials, driven by advances in computational techniques and the development of dispersion‐corrected functionals [8]. Using systematically convergent basis sets, DFT methods can now predict lattice energies with remarkable accuracy, which is crucial for determining sublimation enthalpies [9, 10]. As the primary contributor to the solid‐gas phase transition, the lattice energy plays a central role in accurately estimating sublimation enthalpies. Recent benchmark studies show that dispersion‐corrected DFT may approach sub‐kJ·mol^−1^ accuracy for very small molecular crystals (e.g., benzene) [9, 11], whereas for broader benchmark sets of molecular crystals, typical deviations are several kJ·mol^−1^ (often on the order of ∼4–7 kJ·mol^−1^) [12, 13] with larger errors observed for more complex systems. Despite ongoing challenges, such as properly accounting for electron correlation effects and modeling structural defects, recent advancements, including the development of hybrid functionals and refined thermal correction techniques, have substantially improved the predictive power of DFT [14, 15]. These improvements are increasingly narrowing the gap between theoretical predictions and experimental measurements [16, 17].

In this study, periodic DFT calculations were carried out using the dispersion‐corrected PBE‐D3 functional together with hard projector‐augmented‐wave (PAW) pseudopotentials to determine the sublimation enthalpies of potassium bicarbonate, potassium carbonate, and potassium formate. This substance system is particularly interesting from a technical standpoint because the reaction of formate salts with water to form bicarbonates and hydrogen is a method intensively discussed for hydrogen storage. A detailed description of the computational methodology and experimental procedures is provided below and in the Supporting Information.

## 2 Materials and Methods

### 2.1 Materials

Potassium formate (98.8 wt% purity) and potassium bicarbonate (99.7 wt% purity) were obtained from Carl Roth. Prior to the solution calorimetry experiments, any possible traces of water in both samples were removed by storing them in a vacuum (100 Pa) desiccator with P_2_O_5_ as a moisture absorber.

### 2.2 Solution Calorimetry Measurements

In this work, the enthalpy of formation was determined by using the solution calorimetry technique. Molar enthalpies of the solution of potassium bicarbonate and potassium formate were measured with the modified and computerized commercial LKB 8700‐2 isoperibol solution calorimeter. The detailed description of the calorimeter has been provided in recent publications [18, 19]. Shortly, the dissolution procedure consists of several steps. The 25 mL glass calorimetric cell was filled with 25.00 ± 0.01 g of distilled water. The water amount was determined by weighing using a Sartorius SECURA225D‐1S balance with a reproducibility of 0.0001 g. The sample powder was loaded into a preweighted crushable glass ampoule in a glovebox with a residual gas moisture of 0.2 ppm. The ampoule was closed with a silicon stopper and weighed again to determine the mass of the sample. Additional cell weighing was carried out directly prior to the dissolution experiment to check for possible stopper leakage. All sample weighings were made with a Sartorius balance model MSE3.6P‐000‐DM microbalance with the uncertainty of 5·10^−6^ g. The sample size was selected to obtain the final molality of the solution in the calorimetric cell below 0.03 mol·kg^−^^1^ and to reveal any possible concentration dependence.

Before the experiments with potassium bicarbonate and potassium formate, the calorimeter performance was verified using potassium chloride. The total duration of the experiments, including calibration before breaking the ampoule, dissolution of potassium chloride, and calibration after dissolution, ranged from 1330 to 1480 s. For potassium formate, the duration of the experiments ranged from 1390 to 1420 s. Due to the low solubility of potassium bicarbonate, the length of the main period of the dissolution experiment was increased by adding from 5 to 30 min. to ensure complete dissolution of the sample. The total duration of individual experiments for potassium bicarbonate was 1360, 1665, 1955, 1985, and 2304 s.

### 2.3 Quantum‐Chemical Calculations

To validate the experimentally determined standard enthalpies of formation in the solid phase, ${\text{Δ}}_{f}H_{m}^{o}(\mathrm{cr})$, quantum‐chemical calculations are employed to determine the standard enthalpy of formation in the gas phase, ${\text{Δ}}_{f}H_{m}^{o}(g)$, and the enthalpy of sublimation, ${\text{Δ}}_{\mathrm{cr}}^{g}H_{m}^{o}$. The difference between these two values gives the standard enthalpy of formation in the solid phase:

(1) $${\text{Δ}}_{f}H_{m}^{o}\left(\mathrm{cr}\right)={\text{Δ}}_{f}H_{m}^{o}\left(g\right)-{\text{Δ}}_{\mathrm{cr}}^{g}H_{m}^{o}$$

The enthalpies of formation in the gas phase were derived from an atomization procedure [20] using G4 enthalpies [21] calculated with the Gaussian 16 program package [22]. For every potassium salt, only one stable conformer was found with CREST [23]. The sublimation enthalpy, defined as the difference between the enthalpy of the gaseous phase (*H*_g_) and the molar enthalpy of the crystalline phase (*H*_cr_/*n*), can be expressed as:

(2) $${\text{Δ}}_{\mathrm{cr}}^{g}H_{m}^{o}=H_{g}-H_{\mathrm{cr}}/n=-E_{\mathrm{latt}}+\text{Δ}H_{\mathrm{corr}}$$

where the lattice energy, $E_{\mathrm{latt}}$, which has the dominant contribution to the sublimation enthalpy, represents the electronic energy difference between the crystal and isolated molecules ($E_{\mathrm{latt}}=E_{\mathrm{el},g}-H_{\mathrm{el},\mathrm{cr}}/n$) and $\text{Δ}H_{\mathrm{corr}}$ includes thermal contributions, mainly the zero‐point vibrational energy difference ($\text{Δ}E_{\mathrm{ZPE}}=E_{\mathrm{ZPE},g}-E_{\mathrm{ZPE},\mathrm{cr}}$) and other minor thermal correction terms approximated often by the ideal gas model (see Refs. [9, 11, 24]).

In this study, lattice energies of the crystalline salts and subsequently their sublimation enthalpy were obtained via periodic DFT implemented in the Vienna Ab initio Simulation Package (VASP 6.5.1) [25, 26, 27, 28, 29]. Crystalline structures were optimized using PBE [30] functional along with D3(BJ) dispersion correction [31, 32, 33, 34, 35] and PAW pseudopotentials [29, 36] along an energy cut‐off of 1000 eV. The minimum‐energy volume was determined by scaling the relaxed unit cell (0.85–1.25 V0, 17 points), relaxing the structure at each volume, and fitting the resulting *E*(V) curve to the Murnaghan equation of state. Subsequently, harmonic vibrational frequencies at this volume were computed using the finite displacement method implemented in Phonopy to obtain thermal corrections [37, 38, 39]. For the gas phase, monomers were placed in large periodic cells (30 Å^3^) to avoid intermolecular interactions. The thermal corrections to the enthalpy of the gas phase were calculated at the *Γ*‐point using VASPKIT at 298 K and 1 atm [40]. Further details on the computation methodology are provided in the Supporting Information.

## 3 Results and Discussion

### 3.1 Experimentally Determined Enthalpies of Solution

To determine the solution enthalpies, potassium carbonate and potassium formate were dissolved in deionized water. Due to the large quantity of water present, the reaction equilibrium heavily favors the formation of aquated ions. No solid residues were observed in the experiments. The sample masses varied to get the final concentration in the interval 0.002–0.17 mol⋅kg^−1^ for potassium formate and 0.01–0.03 mol⋅kg^−1^ for potassium bicarbonate to investigate possible concentration‐dependent effects. However, no influence of the concentration on the solution enthalpy was observed, and the enthalpies were calculated as the mean value of all measurements. The results of all individual solution calorimetry experiments are given in Table 1.

**TABLE 1:** Enthalpies of solution at 298.15 K for potassium salts.

Potassium formate		Potassium bicarbonate	
Molality, mol⋅kg^−1^	Δ*H*_sol_ (total), J·mol^−1^	Molality, mol⋅kg^−1^	Δ*H*_sol_ (total), J·mol^−1^
0.00495	1775.9	0.01373	21917
0.00308	2167.1	0.02156	22254
0.00185	1519.3	0.03140	21532
0.01424	1773.4	0.01251	23130
0.10193	1864.7	0.02591	22174
0.17418	1993.7	—	—
Mean^a^	**1850 ± 90**	Mean	**22 200 ± 260**

a Errors are given as the standard error of the mean. The mean values are given in bold.

**SCHEME 1:**
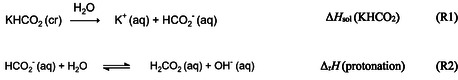
Solution process of potassium formate in water.

**SCHEME 2:**
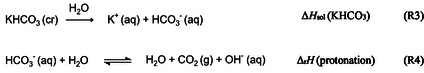
Solution process of potassium bicarbonate in water.

Strictly speaking, the enthalpies measured for potassium formate and potassium carbonate in water include the dissociation of the salt ions, the formation of the hydrate shells, and a certain extent of protonation of the anions (see Schemes 1 and 2).

The calculation of the influence of the protonation reaction on the enthalpy of the solution was provided using the potassium formate protonation as an example. The measured enthalpy, $\text{Δ}H_{\mathrm{sol}}(\text{total})$, is a combination of the solution enthalpy of potassium formate, $\text{Δ}H_{\mathrm{sol}}({\text{KHCO}}_{2})$, and the enthalpy of formate anion protonation, ${\text{Δ}}_{r}H(\text{protonation})$, which is shown in Equation (3):

(3) $$\text{Δ}H_{\mathrm{sol}}(\text{total})=\text{Δ}H_{\mathrm{sol}}({\text{KHCO}}_{2})+\text{α}\cdot {\text{Δ}}_{r}H(\text{protonation})$$

where *α* is the degree of formate protonation. The reaction enthalpy of protonation can be calculated according to the law of Hess and the data from Table S2. The degree of protonation *α* in Equation (3) is determined based on equilibrium calculations. The concentrations of the hydroxide ion, $c({\mathrm{OH}}^{-})$, the formate ion, $c({{\mathrm{HCO}}_{2}}^{-})$, and the undissociated formic acid, $c(H_{2}{\mathrm{CO}}_{2})$, are linked via the base constant, $K_{b}$, in Equation (4):

(4) $$K_{b}=\frac{c\left({\mathrm{OH}}^{-}\right)\cdot c\left(H_{2}{\mathrm{CO}}_{2}\right)}{c\left({{\mathrm{HCO}}_{2}}^{-}\right)}$$

The hydroxide ion and formic acid concentrations are equal and can be calculated as the product of the protonation degree and the initial concentration of formate ions, $c{\left({{\mathrm{HCO}}_{2}}^{-}\right)}^{o}$, with Equation (5):

(5) $$c\left({\mathrm{OH}}^{-}\right)=c\left(H_{2}{\mathrm{CO}}_{2}\right)=\alpha \cdot c{\left({{\mathrm{HCO}}_{2}}^{-}\right)}^{o}$$

In the same way, the equilibrium concentration of the formate ions can be calculated using Equation (6):

(6) $$c\left({{\mathrm{HCO}}_{2}}^{-}\right)=\left(1-\alpha \right)\cdot c{\left({{\mathrm{HCO}}_{2}}^{-}\right)}^{o}$$

Finally, Equations (6) and (7)  can be substituted into Equation (5) to calculate the protonation degree using Equation (7):

(7) $$K_{b}=\frac{c{\left({{\mathrm{HCO}}_{2}}^{-}\right)}^{o}\cdot \alpha^{2}}{1-\alpha }\;\Leftrightarrow \;0=\alpha^{2}+\frac{K_{b}}{c{\left({{\mathrm{HCO}}_{2}}^{-}\right)}^{o}}\cdot \alpha -\frac{K_{b}}{c{\left({{\mathrm{HCO}}_{2}}^{-}\right)}^{o}}$$

Assuming complete dissociation of potassium formate, the initial concentration of the formate ion, $c{\left({{\mathrm{HCO}}_{2}}^{-}\right)}^{o}$, is equal to the concentration of potassium formate in Tables 2 and 3. With these values, the true enthalpy of solution of the potassium salts can be calculated. The results are presented in Table 2 for potassium formate and in Table 3 for potassium bicarbonate.

**TABLE 2:** Degree of formate protonation and true enthalpies of solution of potassium formate at *T *= 298.15 K and p=1bar.

pKa (H_2_CO_2_)	*ρ* (H_2_O), kg⋅l^−1^	c(HCO_2_^−^)^o^, mol⋅l^−1^	*α*	Δ*H*_sol_ (total), J·mol^−1^	Δ_r_ *H* (prot.), J·mol^−1^	Δ*H*_sol_ (KHCO_2_), J·mol^−1^
3.76 ± 0.01	0.997	4.95E‐03	1.08E‐04	1775.9	146780	1760.2
[41]	[42]	3.07E‐03	1.37E‐04	2167.1	—	2147.3
—	—	1.84E‐03	1.77E‐04	1519.3	—	1493.6
—	—	1.42E‐02	6.38E‐05	1773.4	—	1764.2
—	—	1.02E‐01	2.38E‐05	1864.7	—	1861.3
—	—	1.73E‐01	1.82E‐05	1993.7	—	1991.1
—	—	—	—	—	—	—
—	—	—	—	—	Mean	**1840 ± 90**

*Note:* The mean values are given in bold.

**TABLE 3:** Degree of bicarbonate protonation and true solution enthalpies of potassium bicarbonate at *T* = 298.15 K and p=1 bar.

pKa (H_2_CO_3_)	*ρ* (H_2_O), kg⋅l^−1^	c(HCO_3_^−^)^o^, mol⋅l^−1^	*α*	Δ*H*_sol_ (total), J·mol^−1^	Δ_r_ *H* (prot.), J·mol^−1^	**Δ*H***_**sol**_ **(KHCO**_**3**_ **)**, **J·mol** ^**−1**^
6.34 ± 0.01	0.997	1.37E‐02	1.26E‐03	21917	157320	21746
[43]	[42]	2.15E‐02	1.01E‐03	22254	—	22117
—	—	3.13E‐02	8.36E‐04	21532	—	21419
—	—	1.25E‐02	1.32E‐03	23130	—	22952
—	—	2.58E‐02	9.20E‐04	22174	—	22050
—	—	—	—	—	—	—
—	—	—	—	—	Mean	**22060 ± 260**

*Note:* The mean values are given in bold.

The difference between the solution enthalpies of the salts and the total solution enthalpies is roughly 0.1 to 0.2 kJ·mol^−1^. This finding means that the small degree of anion protonation has no significant influence on the enthalpies of formation that are calculated in the next section. Nevertheless, the formation enthalpies are calculated based on the true enthalpies of solution of the potassium salts.

### 3.2 Enthalpies of Formation and Sublimation

With the salts’ true solution enthalpies from Tables 2 and 3 and the ions’ enthalpies of formation in aqueous solution from Table S2, the formation enthalpies of the potassium salts in the solid phase can be calculated with Hess's Law. The results are collected in Table 4. This table also presents the standard enthalpies of formation in the gas phase, as obtained from the G4 calculations, along with the sublimation enthalpies derived from the DFT calculations.

**TABLE 4:** Standard enthalpies of formation and sublimation of potassium salts at 298.15 K in kJ·mol^−1^.

Substance	Δ_f_ $H_{m,G4}^{o}$(g)/ G4	$\Delta_{\mathrm{cr}}^{g}H_{m,\text{DFT}}^{o}$/ DFT	Δ_f_ $H_{m,\exp}^{o}$(cr)/ G4‐DFT	Δ_f_ $H_{m,\exp}^{o}$(cr)/ SC^a^	Δ^b^
Potassium formate	—	—	—	−681.5 ± 5.0 [44]	—
—	−498.0 ± 5.0	178.9 ± 10.0	−676.9 ± 15.0	**−679.5 ± 0.5**	2.6
—	—	—	—	—	—
Potassium bicarbonate	—	—	—	−964.3 ± 5.0 [45]	—
—	—	—	—	−958.0 ± 5.0 [46]	—
—	−743.4 ± 5.0	223.0 ± 10.0	−966.4 ± 15.0	**−964.1 ± 0.3**	2.3
—	—	—	—	—	
Potassium carbonate	—	—	—	−1152.5 ± 5.0 [47]	—
—	—	—	—	−1152.7 ± 5.0 [48]	—
—	—	—	—	−1149.1 ± 5.0 [49]	—
—	—	—	—	−1158.4 ± 5.0 [50]	—
—	−791.9 ± 5.0	351.0 ± 10.0	−1142.9 ± 15.0	−1153.1 ± 2.1 [51]	10.2

a Bold values have been determined with solution enthalpies from this work.

b Difference between columns 4 and 5.

The quantitative assessment of uncertainties provides the basis for validating standard enthalpies of formation in the solid phase. Uncertainties of the G4 calculated enthalpies of formation correspond to the root mean square deviation from experimental data collected for the G3/05 test set from Curtiss et al. [21] Acknowledging the inherent uncertainties associated with calculating sublimation enthalpies using DFT methods is essential. While periodic DFT calculations employing the dispersion‐corrected PBE‐D3 functional and hard PAW pseudopotentials provide valuable insights, the accuracy of predicted sublimation enthalpies is influenced by several factors. Benchmark studies have reported uncertainties of about 6–10 kJ·mol^−1^ for such approaches [15, 24, 52, 53]. These uncertainties stem from the choice of functional and, more generally, the level of theory: for instance, PBE‐D3 may not fully capture all dispersion interactions. Our benchmark test on lithium fluoride (LiF), a prototypical ionic solid with well‐established experimental data, supports this observation (see Supporting Information). Further uncertainty arises from the treatment of vibrational contributions, where inaccuracies in phonon modes and vibrational frequencies can affect thermal corrections [54]. Moreover, the quality of crystal structure input, including lattice parameters and atomic positions, directly impacts the computed values. Future progress will likely come from improved dispersion models beyond D3(BJ), more accurate treatments of vibrational contributions (e.g., anharmonic corrections), and standardized protocols for structure optimization, combined with systematic experimental benchmarking. Together, these developments should enable more reliable predictions of sublimation enthalpies by DFT.

When evaluating the standard enthalpy of formation in the solid phase, a thorough comparison with existing literature data is essential. The enthalpy of formation in the solid phase for potassium carbonate has been reported before in literature and included in thermochemical data collections [51]. The data in it is critically selected and tested for self‐consistency. At the same time, the main references for the studied compounds date back to the last quarter of the 19th century, including the pioneering works of Marcellin Berthelot. Consequently, all data on potassium formate, bicarbonate, and carbonate published to date have been systematically reviewed. The literature data for the enthalpies of solution are summarized in Table S6. Uncertainties were not given in the literature of the 19th century. The uncertainties given for the enthalpies in the crystalline phase calculated with these values were estimated to be 5.0 kJ·mol^−1^. It was found that, with one exception, the available solution enthalpy data originate from the late 19th and early 20th centuries. The most recent source is the study by Benjamin from 1962 [50]. The standard enthalpies of formation in the solid phase were calculated based on Hess's law, using the enthalpies of solution from Table 1 as well as literature data from Table S6 and the standard enthalpies of formation of the ions provided in Table S2. Despite the historical origin of the literature data, all experimentally determined values reported between 1873 and 2025 are in agreement within ±5 kJ·mol^−1^. Given the good agreement between the experimentally and theoretically determined solid‐phase enthalpies of formation, these values are employed in the subsequent section for further calculations of reaction enthalpies.

### 3.3 Reaction Enthalpies

**SCHEME 3:**
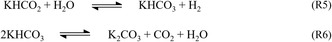
Possible reactions of potassium salts.

For industrial applications, knowledge of the reaction enthalpy is a crucial parameter. These values can be calculated using standard enthalpies of formation and solution, which have been determined in the present work. Two examples of industrially relevant reactions of potassium salts are shown in Scheme 3.

**SCHEME 4:**
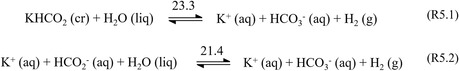
Potassium formate bicarbonate reaction equilibrium for hydrogen storage.

Reaction 5 represents the reaction equilibrium for hydrogen storage and release using the potassium formate–potassium bicarbonate system. Reaction 6 is used industrially for the production of potassium carbonate from potassium bicarbonate. As formulated in Scheme 3, however, the reactions do not yet allow for the calculation of reaction enthalpies. The specification of the physical states of the reactants and products is still missing. For technical applications, the potassium salts can be used either in solid form or dissolved in water. For the potassium formate–bicarbonate system, both of these options are shown in Scheme 4.

In contrast to liquid organic hydrogen carriers, the reaction enthalpy for hydrogen release from potassium formate and water is remarkably low. For commonly used organic compounds such as benzyltoluene and dibenzyltoluene, the dehydrogenation enthalpy at 298.15 K is ≈63‐65 kJ·mol^−1^ per mole of hydrogen [55]. In comparison, the reaction enthalpy of the potassium formate–potassium bicarbonate system at the same temperature is only about 20 kJ·mol^−1^ per mole of hydrogen (see Scheme 4, R5.1 and R5.2). Owing to the low solution enthalpy of potassium formate (≈2 kJ·mol^−1^), it is of negligible relevance for the enthalpy of reaction whether the potassium formate is initially used as a solid (23 kJ·mol^−1^) or in its aquated form in solution (21 kJ·mol^−1^). However, for the overall energy balance of a hydrogen storage process, the presence of water is not negligible, as it has to be heated to the reaction temperature, which significantly contributes to heat demand [56].

**SCHEME 5:**
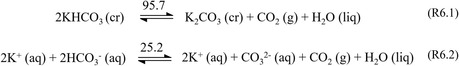
Potassium formate bicarbonate reaction equilibrium for hydrogen storage.

In contrast to R5, the situation is different for reaction 6 (see Scheme 5).

A substantial difference is observed in the formation of potassium carbonate from potassium bicarbonate, depending on whether the bicarbonate is present in solid or dissolved form. The formation of potassium carbonate from solid potassium bicarbonate at 298.15 K is associated with a high reaction enthalpy of 95.7 kJ·mol^−1^ (see Scheme 5, R6.1). Admittedly, the solid potassium carbonate would be partially hydrated by the water produced during the reaction. However, estimating the hydration enthalpy under such high salt concentrations is challenging and would require a dedicated study. Nevertheless, the reaction enthalpy in the aquated phase is significantly lower (25.2 kJ·mol^−1^) (see scheme 5, R6.2). Finally, it must be taken into account that, for the technical production of potassium carbonate, the solvent water would still need to be evaporated. Depending on the amount of water used, the associated energy demand could be considerably higher than that required for the direct thermal decomposition of potassium bicarbonate.

## 4 Conclusion

This work aimed to develop and validate an approach for determining the standard enthalpies of formation and sublimation of salts. Potassium formate, potassium bicarbonate, and potassium carbonate were selected as representative compounds. The standard enthalpies of formation in the solid phase were determined experimentally using solution calorimetry, while the corresponding gas‐phase formation enthalpies were calculated at the G4 level of theory. For salts, the typical approach of using vapor pressure‐derived sublimation enthalpies to bridge the solid and gas phases is not feasible due to their negligible vapor pressures. Therefore, an alternative strategy was employed in this study, using DFT‐based calculations to establish the link between the condensed and gas phases. The validity of this approach was successfully demonstrated using LiF as a reference compound. For the potassium salts, good agreement was found between the experimentally determined standard enthalpies of formation in the solid phase, literature data, and values derived from quantum‐chemical calculations. The combination of DFT calculations and solution calorimetry thus represents a promising approach for the systematic determination of standard formation and sublimation enthalpies of salts in future work.

## Supporting Information

Additional supporting information can be found online in the Supporting Information section. Provenance and purity of the materials, literature data to calculate enthalpies of formation, additional information for quantum‐chemical calculations. **Supporting Table S1:** Provenance and purity of the materials. **Supporting Table S2:** Compilation of enthalpies of formation in the aqueous phase at 298.15 K. **Supporting Table S3:** G4 calculated gas‐phase enthalpies of formation $\Delta_{f}H_{m}^{o}$(g) at T = 298.15 K and p° = 0.1 MPa (in kJ·mol^−1^). **Supporting Table S4:** Lattice energies, ZPE corrections, and sublimation enthalpies for KHCO_2_, KHCO_3_, and K_2_CO_3_ obtained using the PBE‐D3 functional with PAW pseudopotentials at 0 K and 298.15 K (in kJ·mol^−1^). **Supporting Table S5:** DFT calculated sublimation enthalpies for LiF using PBE‐D3 functional with def2‐TZVP and pob‐TZVP (GTO) basis sets and PAW pseudopotential, along with the absolute deviation from the experimental value (276.14 kJ·mol^−1^). **Supporting Table S6:** Standard enthalpies of formation calculated with literature data in kJ·mol^−1^. **Supporting Table S7:** Enthalpies of solution at 298.15 K for potassium chloride.

## Author Contributions

The manuscript was written through the contributions of all authors. All authors have approved the final version of the manuscript. **Zohreh Amanollahi**: investigation, validation, writing. **Dzmitry H. Zaitsau**: investigation, validation. **Karsten Müller**: validation. **Olga S. Bokareva**: conceptualization, investigation, validation, writing – original draft. **Riko Siewert**: supervision, Conceptualization, investigation, validation, writing – original draft.

## Funding

This study was supported by Leibniz‐Gemeinschaft (ID0EP5AE11301), Deutsche Forschungsgemeinschaft (GRK 2943 and 507189291).

## Conflicts of Interest

The authors declare no conflicts of interest.

## Supporting information

Supplementary Material

## Data Availability

The data that support the findings of this study are available in the supplementary material of this article.
