# Supplementary material for: Determination and Validation of Standard Enthalpies of Formation and Sublimation of Potassium Salts Using Solution Calorimetry and Quantum‐Chemical Calculations
Source: ChemistryOpen. 2026 Feb 8;15(2):e202500502. doi: 10.1002/open.202500502 (PMC12883556; doi:10.1002/open.202500502)
Supplement: Supplementary file 1 — Supplementary Material [file OPEN-15-e202500502-s001.pdf]

Electronic Supporting Information

Determination and validation of standard enthalpies of formation and sublimation of potassium salts using solution calorimetry and quantum chemical calculations

Zohreh Amanollahi,[a,b] Dzmitry H. Zaitsau,[b,c] Karsten Müller,[b,c] Olga S. Bokareva,[a,b,d] Riko Siewert [b,c]

[a] Leibniz Institute for Catalysis (LIKAT), Albert-Einstein-Str. 29A, 18059 Rostock, Germany

[b] Department Life, Light & Matter of the Faculty of Interdisciplinary Research at University of Rostock, 18059, Rostock, Germany

[c] Institute of Technical Thermodynamics, University of Rostock, 18059 Rostock, Germany

[d] Institute of Chemistry, University of Rostock, 18059 Rostock, Germany

Table S1. Provenance and purity of the materials.

Materials	CAS	Origin	LOT	Purification	Purity, weight fraction
Potassium formate (cr)	590-29-4	Carl Roth	382328730	Vacuum + P_2O_5	0.992
Potassium bicarbonate (cr)	298-14-6	Carl Roth	322320064	Vacuum + P_2O_5	0.997
Water (l)[a]	7732-18-5	lab		Ultrafiltration, deionization	0.998

[a] The purity of water is assessed using 1.8 μS/cm conductivity provided by the deionization setup.

Solution calorimetry: enthalpy of solution determination

Table S2. Compilation of enthalpies of formation in the aqueous phase at 298.15 K.

Substance	$\Delta_f H^o_{m,\,exp}$(liq)/ kJ·mol^{-1}	$\Delta_f H^o_{m,\,exp}$(g)/ kJ·mol^{-}	$\Delta_{sol} H^{FA/H_2O\,b}$ kJ·mol^{-1}	$\Delta_f H^o_m$ (aq)/ kJ·mol^{-1}
Formic acid	-424.8 ± 0.3 [1]		-0.678 ± 0.001 [2]	-425.5 ± 0.3 [a]
Carbon dioxide		-393.51 ± 0.13 [3]		
Water	-285.83 ± 0.04 [3]			-285.83 ± 0.04 [3]
K^+ (aq)				-252.14 ± 0.08 [4]
HCO_2^- (aq)				-425.48 ± 0.45 [4]
HCO_3^- (aq)				-689.862 ± 0.004 [4]
CO_3^{2-} (aq)				-675.16 ± 0.053 [4]
OH^- (aq)				-139.030± 0.022 [4]

[a] Difference between columns 2 and 4.
[b] infinite dilution in water

Quantum chemical calculations: enthalpies of formation in the gas phase

Table S3. G4 calculated gas-phase enthalpies of formation $\Delta_f H^o_m$(g) at T = 298.15 K and $p°$ = 0.1 MPa (in kJ·mol^{-1}).

Substance	Perspective 1	Perspective 2	H_{298}(G4) [a]	$\Delta_f H^o_m$(g) [b]
Potassium formate	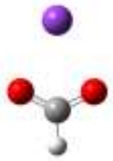	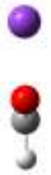	-788.895812	-498.0±5.0
Potassium bicarbonate	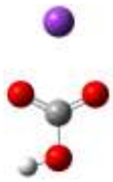	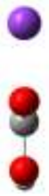	-864.127131	-743.4±5.0
Potassium carbonate	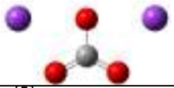	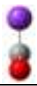	-1463.307246	-791.9±5.0

[a] Calculated with the G4 method [5].
[b] Calculated with $\Delta_f H^o_m$ = 89.885 kJ·mol^{-1} for K(g) at 0 K and H-H^o (T_r) = 7.082 kJ·mol^{-1} according to NIST-JANAF Thermochemical Tables [6] using an atomization reaction [7]

DFT Computational Details

The lattice energies of the crystalline salts and subsequently their sublimation enthalpies were obtained via periodic density functional theory (DFT) implemented in the Vienna Ab Initio Simulation Package (VASP 6.5.1). To do so, we started by optimizing the unit-cell parameters and atomic positions of the experimental crystal structures, which were obtained from the Materials Project ($KHCO_2$: mp-643989, $KHCO_3$: mp-634431, and K_2CO_3: mp-3963).[8] To identify the minimum-energy volume, the initially relaxed unit cell (volume V_0) was systematically scaled from 0.85 to 1.25 V_0 in 17 increments. For each scaled structure, a constant-volume relaxation was performed, allowing the internal coordinates to relax while the unit-cell volume remained fixed. The resulting E(V) data were fitted to the Murnaghan equation of state, and the equilibrium volume was then used as the reference point for lattice energy calculations and subsequent vibrational analysis. Optimizations were performed using the PBE functional with D3(BJ) dispersion correction and PAW pseudopotentials, utilizing an energy cut-off of 1000 eV. Subsequently, harmonic vibrational frequencies at this volume were computed using the finite displacement method implemented in Phonopy to obtain thermal corrections. For the gas phase, monomers were placed in large periodic cells (30 $Å^3$) to avoid intermolecular interactions, and thermal corrections to the enthalpy were calculated at the Γ-point using Vaspkit at 298.15 K and 1 atm. Brillouin-zone integrations were performed using Monkhorst–Pack k-point meshes generated with the Vaspkit utility. A reciprocal-space resolution of 0.03 × 2π $Å^{-1}$ was applied, with meshes centered at the Γ-point. And convergence criteria of EDIFF = 10^{-8} eV and EDIFFG = –0.01 eV/Å were applied. Table SXX summarizes all results obtained from these calculations, including the lattice energies of all crystalline salts, zero-point vibrational energy (ZPE) corrections, and their sublimation enthalpies at 0 K ($\Delta_{cr}^{g}H_m^o\ (0K) = -E_{latt} + \Delta E_{ZPE}$) as well as at 298.15 K.

Table S4. Lattice energies, ZPE corrections, and sublimation enthalpies for $KHCO_2$, $KHCO_3$, and K_2CO_3 obtained using the PBE-D3 functional with PAW pseudopotentials at 0 K and 298.15 K (in kJ·mol^{-1}).

Substance	E_{latt}	ΔE_{ZPE}	$\Delta_{cr}^{g}H_m^o\ (0K)$	$\Delta_{cr}^{g}H_m^o\ (298.15K)$
Potassium formate	-184.74	-3.75	180.99	178.86
Potassium bicarbonate	-226.49	-3.11	223.38	222.97
Potassium carbonate	-356.73	-3.82	352.91	351.02

Benchmark calculations on LiF

To validate the computational approach, sublimation enthalpy calculations were first performed for LiF, a prototypical ionic solid with well-established experimental data. Calculations were carried out with the Turbomole program package,[9] using Gaussian-type orbitals (GTO). Several functionals (PBE-D3, B97-D, TPSS-D3, M06-L[10–13]) and basis sets (def2-TZVP, def2-QZVP, pob-TZVP [14–16]) were tested on the experimental structure (mp-1138). [8] Subsequently, plane-wave DFT calculations using VASP (PBE-D3/PAW) were performed for LiF.

Figure S1 shows the deviation of the calculated sublimation enthalpies from the experimental reference value,[17] across the different functional and basis set combinations. Several key trends were identified: the def2-TZVP basis set consistently produced accurate and well-balanced results across all functionals. Although def2-QZVP yielded slightly smaller deviations, it showed inconsistent performance with different k-point meshes. The def2-SVP basis set significantly overestimated sublimation enthalpies and was therefore deemed unsuitable. The pob-TZVP basis set gave moderately accurate results and aligns with recommendations in the Turbomole manual. Among the functionals tested, B97-D and PBE-D3 offered the best overall performance, followed by TPSS-D3 and M06-L. Both B97-D and PBE-D3 produced deviations within an acceptable range

when combined with def2-TZVP. The effect of k-point mesh density was negligible, especially with the def2-TZVP basis set, as increasing the grid from 3×3×3 to 21×21×21 changed the lattice energy by less than 1 kJ·mol^{-1}.

Comparison of these results with those obtained using the computationally more efficient plane-wave setup in VASP (horizontal red line in Figure S1) shows excellent agreement with the Turbomole PBE-D3/triple-ζ (GTO) results. This comparison confirms that the plane-wave approach is both accurate and computationally efficient, justifying its use for the potassium salts investigated in this study.

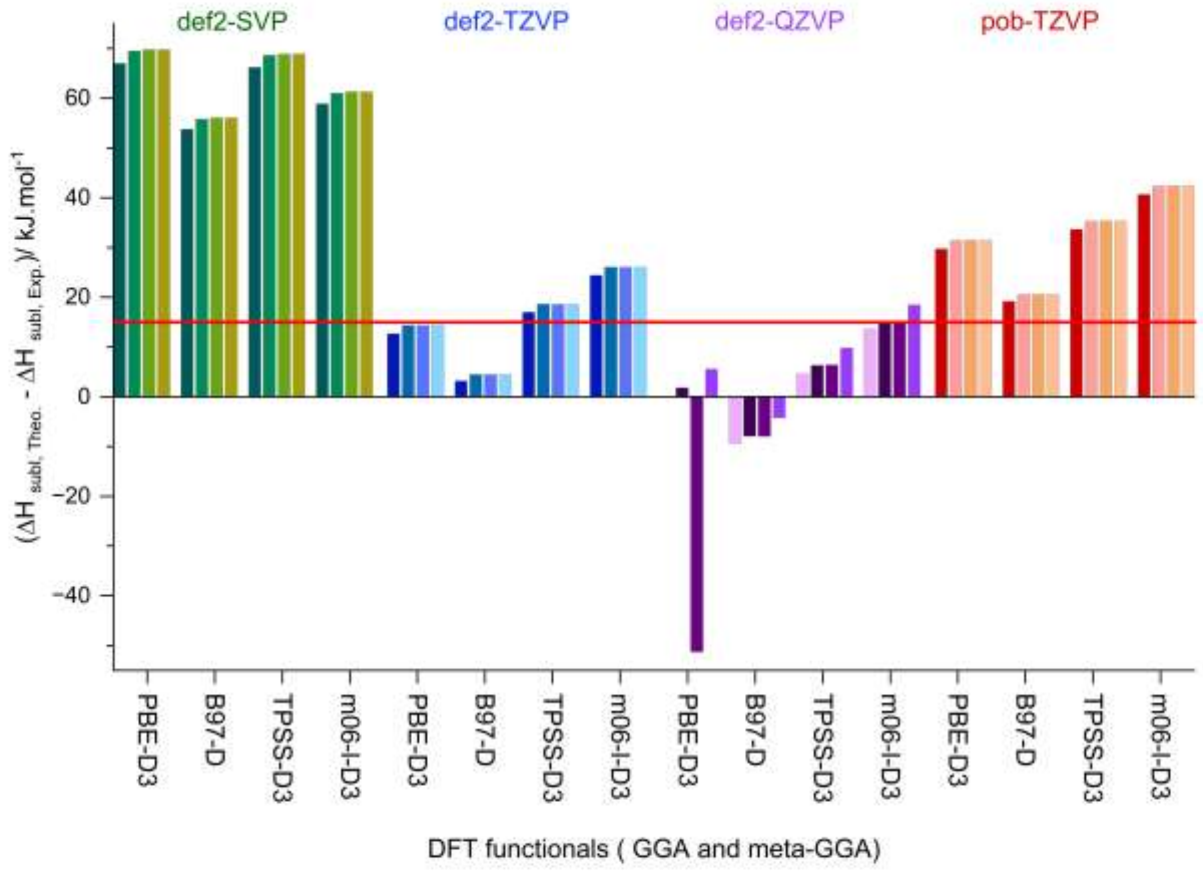

Figure S1. Deviations from the experimental sublimation enthalpy of LiF for different functional/basis set combinations. Each group shows results with increasing k-point density (3×3×3, 5×5×5, 8×8×8, 21×21×21) from left to right. The horizontal red line indicates the value obtained from VASP calculations using the PBE-D3 (PAW).

Table S5. DFT calculated sublimation enthalpies for LiF using PBE-D3 functional with def2-TZVP and pob-TZVP (GTO) basis sets and PAW pseudopotential, along with the absolute deviation from the experimental value (276.14 kJ·mol^{-1}).

Theory level	$\Delta_{cr}^{g}H_{m}^{o}$	$\Delta\Delta_{cr}^{g}H_{m}^{o}$
PBE-D3/ PAW	290.96	14.79
PBE-D3/ def2-TZVP	294.25	18.11
PBE-D3/ pob-TZVP	311.15	35.01

Uncertainty budget evaluation

The calculation of standard deviations was carried out with Eq. S1 for total solution enthalpies:

$$\sigma = \sqrt{\frac{\sum(\mathrm{x}-\bar{\mathrm{x}})^2}{(\mathrm{n}-1)}} \qquad \text{(Eq. S1)}$$

where x is the solution enthalpy of every individual measurement shown in Table 1, and n is the number of individual measurements for every substance. The uncertainty of the total solution enthalpy was then calculated with Eq. S2.

$$\mathrm{u}(\Delta H_{\mathrm{sol}}\ (\mathrm{total})) = \frac{\sigma}{\sqrt{\mathrm{n}}} \qquad \text{(Eq. S2)}$$

The following uncertainties are used in Eq. S3 to calculate the uncertainty of the salts true solution enthalpy, $\mathrm{u}(\Delta H_{\mathrm{sol}}(\mathrm{salt}))$: The uncertainty of the total enthalpy of solution, $\mathrm{u}(\Delta H_{\mathrm{sol}}\ (\mathrm{total}))$, the uncertainties of the standard enthalpies of formation of the dissociated ions in water, $\mathrm{u}\left(\Delta_{\mathrm{f}}H_{\mathrm{m}}^{\mathrm{o}}(\mathrm{aq})\right)$, the uncertainty of the anion protonation degree, $\mathrm{u}(\alpha)$, and the enthalpy of reaction uncertainty for the protonation, $u(\Delta_{\mathrm{r}}H)$:

$$\mathrm{u}(\Delta H_{\mathrm{sol}}(\mathrm{salt})) = \sqrt{\left(\mathrm{u}(\Delta H_{\mathrm{sol}}(\mathrm{total}))\right)^2 + \alpha \cdot \left(\mathrm{u}(\alpha)\right)^2 + \alpha \cdot \left(\mathrm{u}(\Delta_{\mathrm{r}}H)\right)^2} \qquad \text{(Eq. S3)}$$

The protonation degree uncertainty, $\mathrm{u}(\alpha)$, is calculated with Eq. S4.

$$\mathrm{u}(\alpha) = \sqrt{\left(\mathrm{u}(\mathrm{p}K\mathrm{a})\right)^2 + \left(\mathrm{u}(\mathrm{c}(\mathrm{salt})^{\mathrm{o}})\right)^2} \qquad \text{(Eq. S4)}$$

For the calculation of the reaction enthalpy uncertainty, $\mathrm{u}(\Delta_{\mathrm{r}}H)$, the uncertainties of the formation enthalpies of reactants, $\mathrm{u}\left(\Delta_{\mathrm{f}}H_{\mathrm{m}}^{\mathrm{o}}(\mathrm{reactant})\right)$, and products, $\mathrm{u}\left(\Delta_{\mathrm{f}}H_{\mathrm{m}}^{\mathrm{o}}(\mathrm{product})\right)$, from Table S2 are used in Eq. S5.

$$\mathrm{u}(\Delta_{\mathrm{r}}H) = \sqrt{\sum\left(\mathrm{u}\left(\Delta_{\mathrm{f}}H_{\mathrm{m}}^{\mathrm{o}}(\mathrm{reactants})\right)\right)^2 + \sum\left(\mathrm{u}\left(\Delta_{\mathrm{f}}H_{\mathrm{m}}^{\mathrm{o}}(\mathrm{products})\right)\right)^2} \qquad \text{(Eq. S5)}$$

The following uncertainties are used in Eq. S6 to calculate the standard enthalpy of formation uncertainties of salts in the solid phase, $\mathrm{u}\left(\Delta_{\mathrm{f}}H_{\mathrm{m}}^{\mathrm{o}}(\mathrm{cr})\right)$:

$$\mathrm{u}\left(\Delta_{\mathrm{f}}H_{\mathrm{m}}^{\mathrm{o}}(\mathrm{cr})\right) = \sqrt{\left(\mathrm{u}(\Delta H_{\mathrm{sol}}(\mathrm{salt}))\right)^2 + \left(\mathrm{u}\left(\Delta_{\mathrm{f}}H_{\mathrm{m}}^{\mathrm{o}}(\mathrm{salt, aq})\right)\right)^2} \qquad \text{(Eq. S6)}$$

The uncertainties of the formation enthalpy in aqueous solution of the cation, $\mathrm{u}\left(\Delta_{\mathrm{f}}H_{\mathrm{m}}^{\mathrm{o}}(\mathrm{cation, aq})\right)$, and the anion, $\mathrm{u}\left(\Delta_{\mathrm{f}}H_{\mathrm{m}}^{\mathrm{o}}(\mathrm{anion, aq})\right)$¸ are taken from Table S2 and used to calculate the uncertainty of the salt in aqueous solution, $\mathrm{u}\left(\Delta_{\mathrm{f}}H_{\mathrm{m}}^{\mathrm{o}}(\mathrm{salt, aq})\right)$, with Eq. S7.

$$u\left(\Delta_f H_m^o(\text{salt,aq})\right) = \sqrt{\left(u\left(\Delta_f H_m^o(\text{cation,aq})\right)\right)^2 + \left(u\left(\Delta_f H_m^o(\text{anion,aq})\right)\right)^2} \quad \text{(Eq. S7)}$$

Table S6. Standard enthalpies of formation calculated with literature data in kJ·mol^{-1} [a].

Substance	ΔH_{sol} kcal·mol^{-1}	ΔH_{sol} kJ·mol^{-1}	T in K	$\Delta_f H_m^o$(cr) kJ·mol^{-1}	source
Potassium formate	0.93	3.9	288	-681.5	[18]
Potassium bicarbonate	5.32	22.3	288	-964.3	[19]
	3.82 [c]	16.0	290	-958.0	[20]
Potassium carbonate	-6.44	-26.9	288	-1152.5	[21]
	-6.402	-26.8	293	-1152.7	[22]
	-7.24 [d]	-30.3	298	-1149.1	[23]
		-21.0	298	-1158.4	[24]

[a] The exothermic enthalpy of solution in the literature of the 19th century was often given with a positive sign, contrary to today's definition. The literature values were corrected in accordance with today's definition.

[b] Estimated from the typical conditions that Berthelot reported in other solution studies.

[c] enthalpy of neutralization of 6.34 kcal·mol^{-1} given in the literature was corrected by taking into account the standard enthalpies of formation of water, carbon dioxide, and bicarbonate anion given in Table S2.

[d] Solution enthalpy in calories per gram equivalent given as: "3.25 + 0.037 (T – 15)" with T in °C. The gram equivalent was corrected to moles by multiplication with 2. The enthalpy of solution was given with a positive sign, contrary to today's definition. The calculation was therefore carried out as follows: ΔH_{sol}(298 K) = -(3.25 + 0.037) 2 kcal·mol^{-1}

Table S7. Enthalpies of solution at 298.15 K for potassium chloride.

Experiment duration in seconds	Molality, mol·kg^{-1}	ΔH_{sol} J·mol^{-1}
1304	0.00167	17416
1312	0.01153	17412
1323	0.01573	17575
1323	0.00401	17484
1324	0.00652	17709
1324	0.02089	17529
1334	0.01724	17652
1355	0.00781	17536
1364	0.03703	17624
1373	0.03419	17800
1405	0.05704	17710
1417	0.05056	17780
1468	0.08921	17717
1811	0.11217	17634
mean [a]		**17613±33**
reference		17584±17 [25]

[a] The errors are given as the standard error of the mean.

[1] G. C. Sinke, *J. Phys. Chem.* **1959**, *63*, 2063.
[2] J. Konicek, I. Wadso, *Acta Chem. Scand.* **1971**, *25*, 1461–1551.
[3] J. D. Cox, D. D. Wagman, V. A. Medvedev **1989**.
[4] B. Ruscic, D. H. Bross, *Active Thermochemical Tables (ATcT) Thermochemical Values ver. 1.124*.
[5] L. A. Curtiss, P. C. Redfern, K. Raghavachari, *J. Chem. Phys.* **2007**, *126*, 84108.
[6] M. W. Chase, *J. Phys. Chem. Ref. Data* **1998**, 1–1951.
[7] R. Notario, O. Castaño, R. Gomperts, L. M. Frutos, R. Palmeiro, *J. Org. Chem.* **2000**, *65*, 4298–4302.
[8] A. Jain et al., *APL Mater.* **2013**, *1*.
[9] V. Turbomole, *TURBOMOLE GmbH, since 2007;* **2022**.
[10] J. P. Perdew, K. Burke, M. Ernzerhof, *Phys. Rev. Lett.* **1996**, *77*, 3865.
[11] S. Grimme, *J. Comput. Chem.* **2006**, *27*, 1787–1799.
[12] Y. Zhao, D. G. Truhlar, *Theor. Chem. Acc.* **2008**, *120*, 215–241.
[13] J. Tao, J. P. Perdew, V. N. Staroverov, G. E. Scuseria, *Phys. Rev. Lett.* **2003**, *91*, 146401.
[14] F. Weigend, R. Ahlrichs, *Phys. Chem. Chem. Phys.* **2005**, *7*, 3297–3305.
[15] M. F. Peintinger, D. V. Oliveira, T. Bredow, *J. Commput. Chem.* **2013**, *34*, 451–459.
[16] S. Grimme, J. Antony, S. Ehrlich, H. Krieg, *J. Chem. Phys.* **2010**, *132*, 154104.
[17] V. P. Glushko, V. A. Medvedev, *Thermal constants of substances*, New York: Hemisphere Publishing Company, **1990**.
[18] M. Berthelot in *Annales de chimie et de physique* (Eds.: M. E. Chevreul, J.-B. Dumas, J.-B. J. D. Boussingault, H. V. Regnault, C.-A. Wurtz), G. Masson, Paris, **1873**.
[19] R. H. de Forcrand, *C. R. Acad. Sci.* **1909**, *149*, 720.
[20] J. A. Muller in *Annales de chimie et de physique* (Eds.: M. E. Chevreul, M. Berthelot, L. Pasteur, C. Friedel, A. E. Becquerel, É. É. N. Mascart), G. Masson, Paris, **1888**.
[21] W. Ostwald, *J. Prakt. Chem.* **1882**, *25*, 1–19.
[22] R. H. de Forcrand, *C. R. Acad. Sci.* **1909**, *149*, 100.
[23] M. Berthelot, P. Ilovsvay in *Annales de chimie et de physique* (Eds.: M. E. Chevreul, J.-B. A. Dumas, J.-B. Boussingault, C. A. Wurtz, M. Berthelot, L. Pasteur), G. Masson, Paris, **1883**.
[24] L. Benjamin, *J. Chem. Eng. Data* **1962**, *7*, 239–240.
[25] M. V. Kilday, *Journal of research of the National Bureau of Standards (1977)* **1980**, *85*, 467–482.
